# Draft Genome Sequences of Idiomarina abyssalis Strain KJE, Marinobacter salarius Strain NP2017, and Marinobacter salarius Strain AT3901, Isolated from Deep-Sea Sediment near the Western Flank of the Mid-Atlantic Ridge

**DOI:** 10.1128/MRA.01295-20

**Published:** 2021-01-21

**Authors:** Megan M. Mullis, Rachel E. Weisend, Brandi Kiel Reese

**Affiliations:** a Texas A&M University—Corpus Christi, Department of Life Sciences, Corpus Christi, Texas, USA; b Dauphin Island Sea Lab, Dauphin Island, Alabama, USA; c University of South Alabama, Department of Marine Sciences, Mobile, Alabama, USA; Georgia Institute of Technology

## Abstract

We report the draft genomes of environmental cultures collected from shallow sediment from the western flank of the Mid-Atlantic Ridge. The isolates were most closely related to Idiomarina abyssalis strain KJE (100% complete), Marinobacter salarius strain NP2017 (97.6% complete), and Marinobacter salarius strain AT3901 (98.4% complete). Isolates identified as an *Idiomarina* species possess complete nitrite oxidation and reduction pathways, and isolates identified as a *Marinobacter* species possess complete dissimilatory nitrate reduction pathways.

## ANNOUNCEMENT

**FIG 1:**
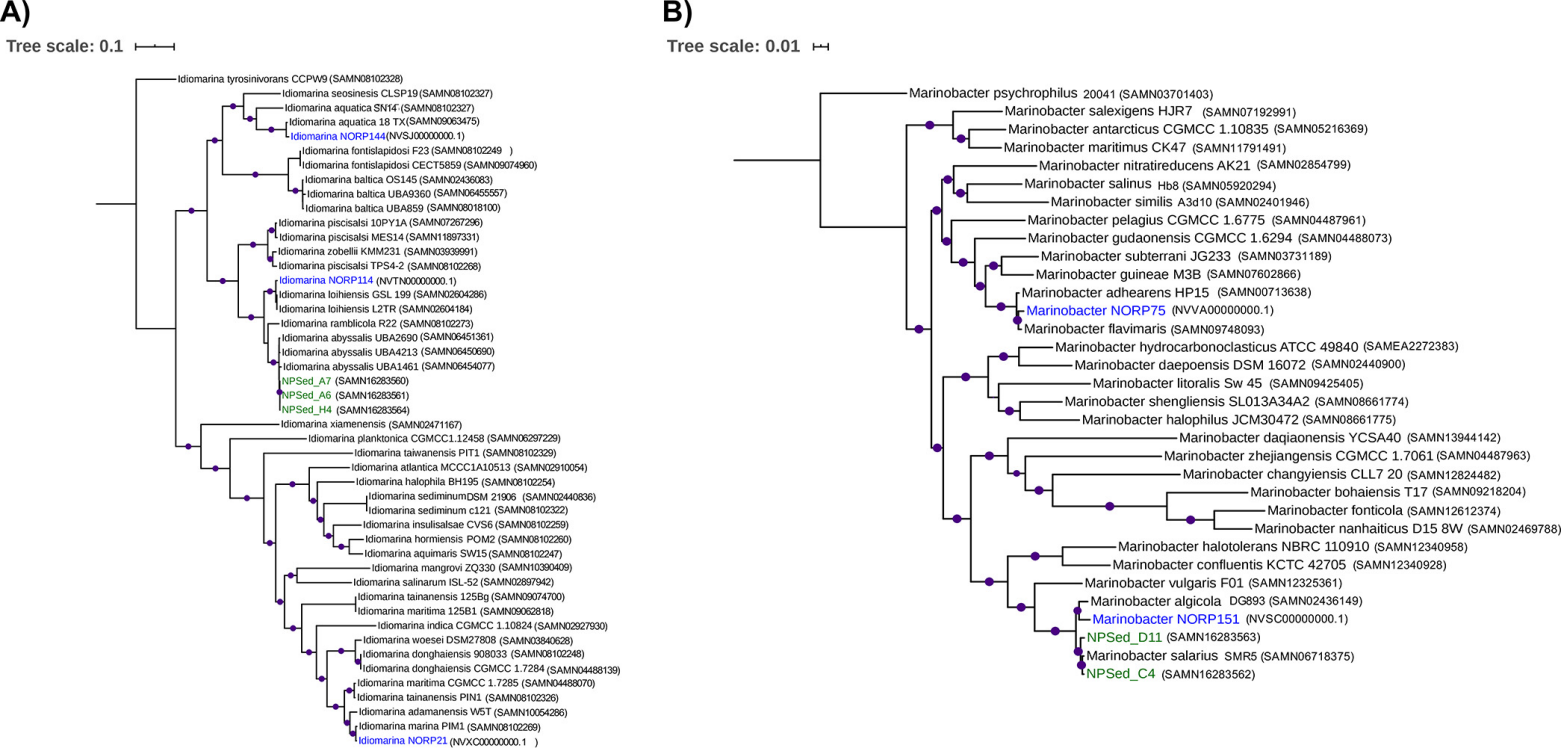
Phylogenetic trees containing all isolates from North Pond. (A) Phylogenetic tree of *Idiomarina*. (B) Phylogenetic tree of *Marinobacter*. These trees were constructed from single-copy marker genes isolated using ezTree v0.1 (default options) (19). The single-copy marker genes were concatenated and aligned using MUSCLE v3.8.31 (default options) (22). The alignment file was used to construct a phylogenetic tree using IQ-TREE v1.6.7.1 with 10,000 bootstraps (-B 10000) (20). The trees were visualized using the iTOL Web server (23). Genomes in blue text are metagenome-assembled genomes previously found in North Pond basaltic fluids (24). The draft genomes from this study are shown in green text. The purple dots signify bootstrap values of ≥90%. The accession number for each reference is located in parentheses after the isolate name.

The western flank of the Mid-Atlantic Ridge, also referred to as North Pond, has oligotrophic sediment dominated by nitrogen-cycling microorganisms (1–3). During an expedition aboard the R/V *Atlantis* (AT3901) in October 2017, we collected the top 1 m of sediment using push cores via the ROV *Jason II*. Aliquots (1 cm^3^) of sediment from 0 to 2 cm and 4 to 6 cm were placed in 10 ml sterile artificial seawater on board, stored at 4°C, and transported on ice to our home laboratory. The sediment was serially diluted into sterile artificial seawater in preparation for sorting. Single cells were sorted into individual wells of a 96-well plate using fluorescence-activated cell sorting (FACSJazz; BD, Franklin Lakes, NJ) and LIVE/DEAD staining to target viable cells. Each well contained 170 ml of sterile artificial seawater amended with vitamins and trace metals, including manganese, zinc, cobalt, molybdenum, selenium, and nickel (4). Two exterior columns of the microtiter plate contained only sterile medium to track contamination. Plates containing individual isolates were incubated at 27°C, and growth was monitored daily through optical density measurements for 12 days. Successfully growing isolates were screened using 16S rRNA gene Sanger sequencing, and five unique isolates were selected for whole-genome sequencing. Selected isolates were grown in triplicate in 75 ml of the same medium at 27°C for 3 to 4 weeks, with agitation every 2 days. The large batch cultures were centrifuged at 5,000 rpm for 10 min at room temperature, the supernatant was decanted, and the pellet was resuspended in 200 μl of PCR-grade water. Nucleic acids were extracted from pellets by heating for 10 min at 100°C and then cleaned and concentrated using the Zymo Clean and Concentrator kit following the manufacturer's protocols (Zymo, Irvine, CA). Libraries were prepared using the Nextera Flex kit (Illumina, San Diego, CA) and sequenced using an Illumina MiSeq system with 300-bp paired-end chemistry at Integrated Microbiome Research (Halifax, Nova Scotia, Canada). Overlapping reads were merged with FLASH v1.2.11 (-M 300) (5). Adapter sequences and low-quality bases were trimmed from merged and unmerged sequences using Trim Galore v0.6.5 (6). Trimmed reads were assembled using SPAdes v3.13.0 (-k 127, --careful) (7) and Velvet v1.2.10 (-k-mer range, 77--137, -k-step 10) (8). Draft assemblies were filtered by length (500-bp cutoff) using SeqKit v0.13.2 (9) and were optimized using QUAST v4.1 (10, 11). Velvet produced the best assemblies for all five isolates, and the assemblies were annotated using the National Center for Biotechnology Information (NCBI) Prokaryotic Genome Annotation Pipeline (PGAP) (12), Prokka v1.11 (13), GhostKOALA (14), and PATRIC v3.6.7 (15) (Table 1). Genome assembly contamination was assessed using CheckM (16) and BUSCO (17). All genome assemblies showed less than 1% contamination, indicating a pure culture for each isolate. Whole-genome assemblies for isolates NPSed_A6, NPSed_A7, and NPSed_H4 were closely related to Idiomarina abyssalis (97.8%, 97.8%, and 97.8% similarity, respectively) and those for NPSed_C4 and NPSed_D11 were closely related to Marinobacter salarius (98.5% and 96.05% similarity, respectively) based on average nucleotide identity (ANI) values determined using JSpeciesWS v3.6.2 (18). JSpeciesWS is a tool that performs pairwise comparisons of ANI values and tetranucleotide signatures of draft genome assemblies (18). Reference genome assemblies for *Idiomarina* and *Marinobacter* genera from GenBank were used for the comparisons. Reference genomes were selected based on genera and completeness of the genomes within GenBank. Each isolate was phylogenetically placed via single-copy marker genes using ezTree v0.1 (19) and IQ-TREE v1.6.7.1 with 10,000 bootstraps (20). Phylogenetic trees were visualized using the Interactive Tree Of Life (iTOL) Web server (Fig. 1). NPSed_A6, NPSed_A7, and NPSed_H4 genomes were 99.9% similar to each other based on ANI values, indicating the same strain. These three isolates are identified as Idiomarina abyssalis strain KJE. NPSed_C4 is identified as Marinobacter salarius strain NP2017, and NPSed_D11 is identified as Marinobacter salarius strain AT3901. GhostKOALA was used to determine the completeness of metabolic pathways with the KEGG-decoder.py script (21). Idiomarina abyssalis strain KJE had complete pathways for nitrite oxidation and reduction (*nxrAB*, *nirK*, and *nirS*). Marinobacter salarius strains NP2017 and AT3901 had complete pathways for dissimilatory nitrate reduction (*narGH* and *napAB*), sulfur assimilation (*sir* and *cysJI*), and sulfur dioxygenase (*sdo*). All genomes possessed complete metabolic pathways for flagellum biosynthesis (*flgABDEFGHILK*, *fliBGHMNOYZ*, and *flhAB*) and motility (*cheABCRVWZY* and *motAB*).

**TABLE 1:** North Pond sediment assembly metrics for the five environmental strains

Strain	Isolate name	Raw sequence accession no.	Genome accession no.	Collection depth (cm)	No. of contigs	G+C content (%)	K-mer size	*N*_50_ (bp)	*L*_50_	Sequence coverage (×)	Genome length (bp)	Genome completeness (single-copy genes) (%)	No. of protein-coding sequences
Idiomarina abyssalis strain KJE-1	NPSed_A6	SAMN16283560	JAEMOO000000000	0–2	45	47	127	108,570	8	180	2,654,914	100	2,575
Idiomarina abyssalis strain KJE-2	NPSed_A7	SAMN16283561	JAEMOP000000000	0–2	9	47	127	1,927,400	1	221	2,861,585	99.2	2,575
Marinobacter salarius strain NP2017	NPSed_C4	SAMN16283562	JAEMOQ000000000	0–2	139	57	127	91,165	12	132	4,359,230	97.6	4,129
Marinobacter salarius strain AT3901	NPSed_D11	SAMN16283563	JAEMOR000000000	4–6	134	57	127	79,996	15	146	4,480,477	98.4	4,303
Idiomarina abyssalis strain KJE-3	NPSed_H4	SAMN16283564	JAEMOS000000000	0–2	74	47	127	81,319	12	192	2,847,554	100	2,840

### Data availability.

These whole-genome assemblies were deposited in GenBank under the accession numbers listed in Table 1. The raw sequences for each isolate were deposited in the Sequence Read Archive (SRA) under BioProject accession number PRJNA666193.
